# Supplementary material for: SILAC-Based Quantitative Proteomic Analysis of Oxaliplatin-Resistant Pancreatic Cancer Cells
Source: Cancers (Basel). 2021 Feb 10;13(4):724. doi: 10.3390/cancers13040724 (PMC7916634; doi:10.3390/cancers13040724)
Supplement: Supplementary file 1 [file cancers-13-00724-s001.zip › Supplementary Material/Supplementary Figures.docx]

Supplementary Metarials: SILAC-Based Quantitative Proteomic Analysis of Oxaliplatin-Resistant Pancreatic Cancer Cells

Young Eun Kim, Eun-Kyung Kim, Min-Jeong Song, Tae-Young Kim, Ho Hee Jang and Dukjin Kang
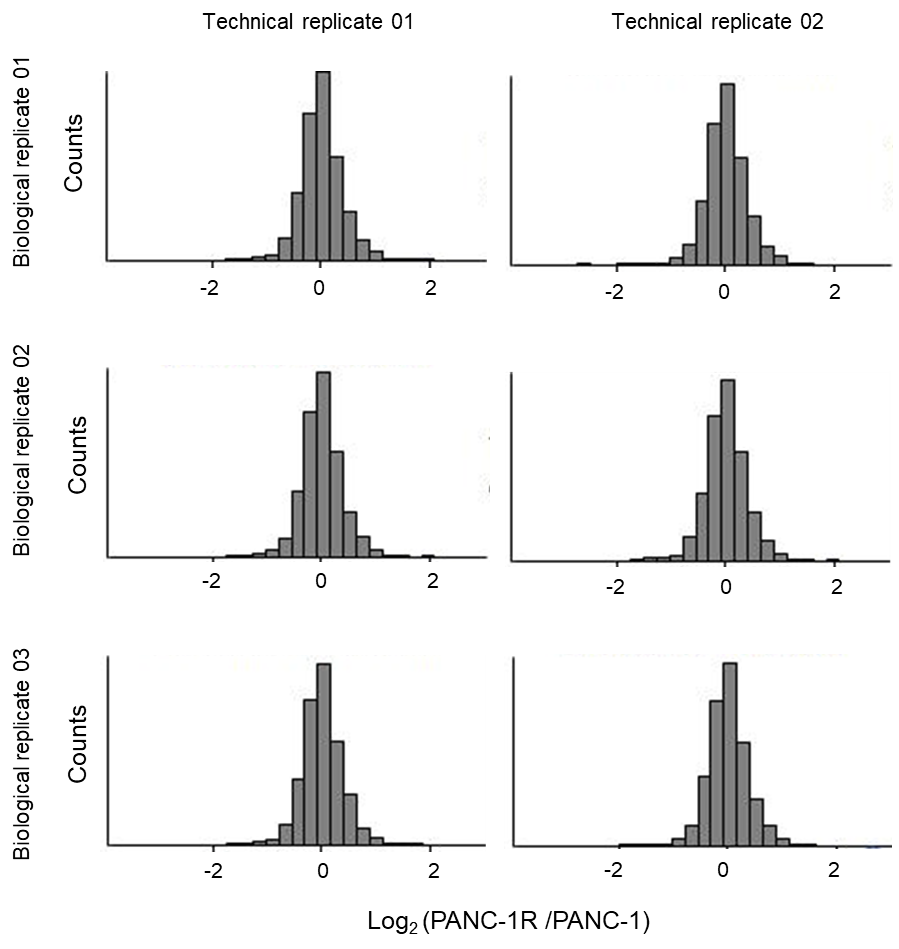


**Figure S1.** Histograms of log_2_ fold-changes for PANC-1R /PANC-1. Three biological replicates were analyzed and two technical repeats were performed for each sample. PANC-1R, oxaliplatin resistant PANC-1 cells. .


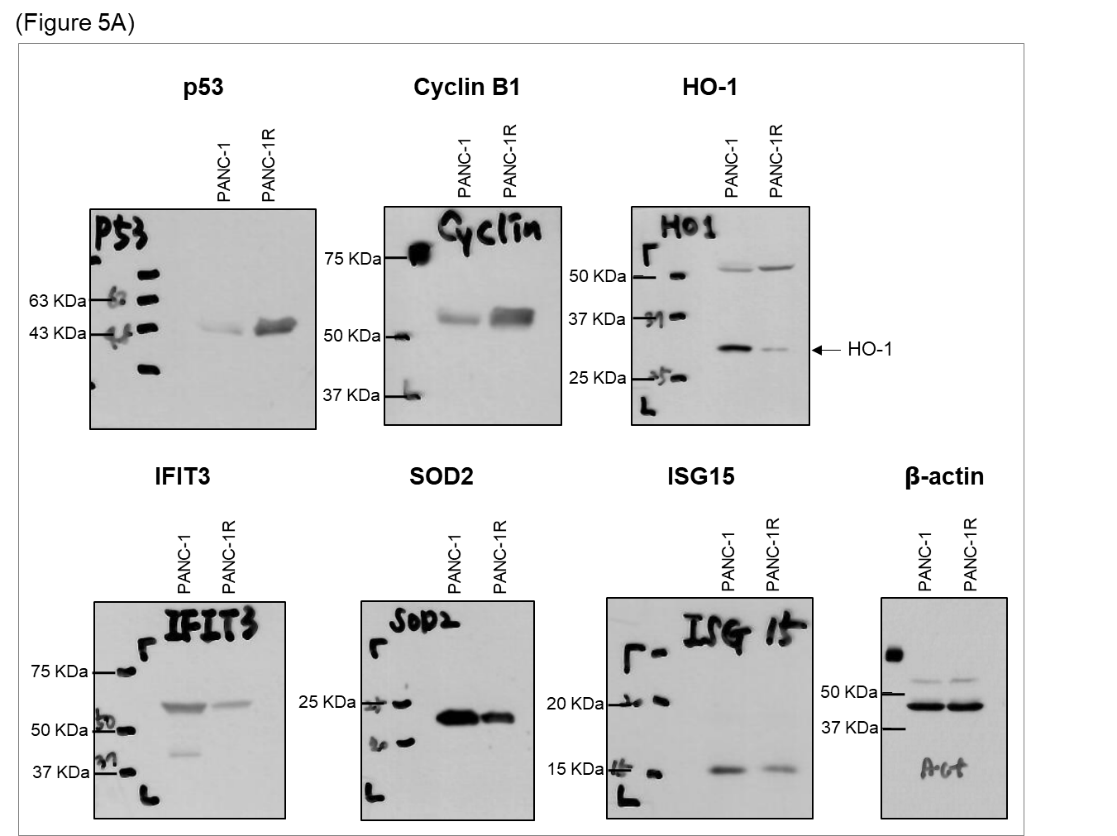


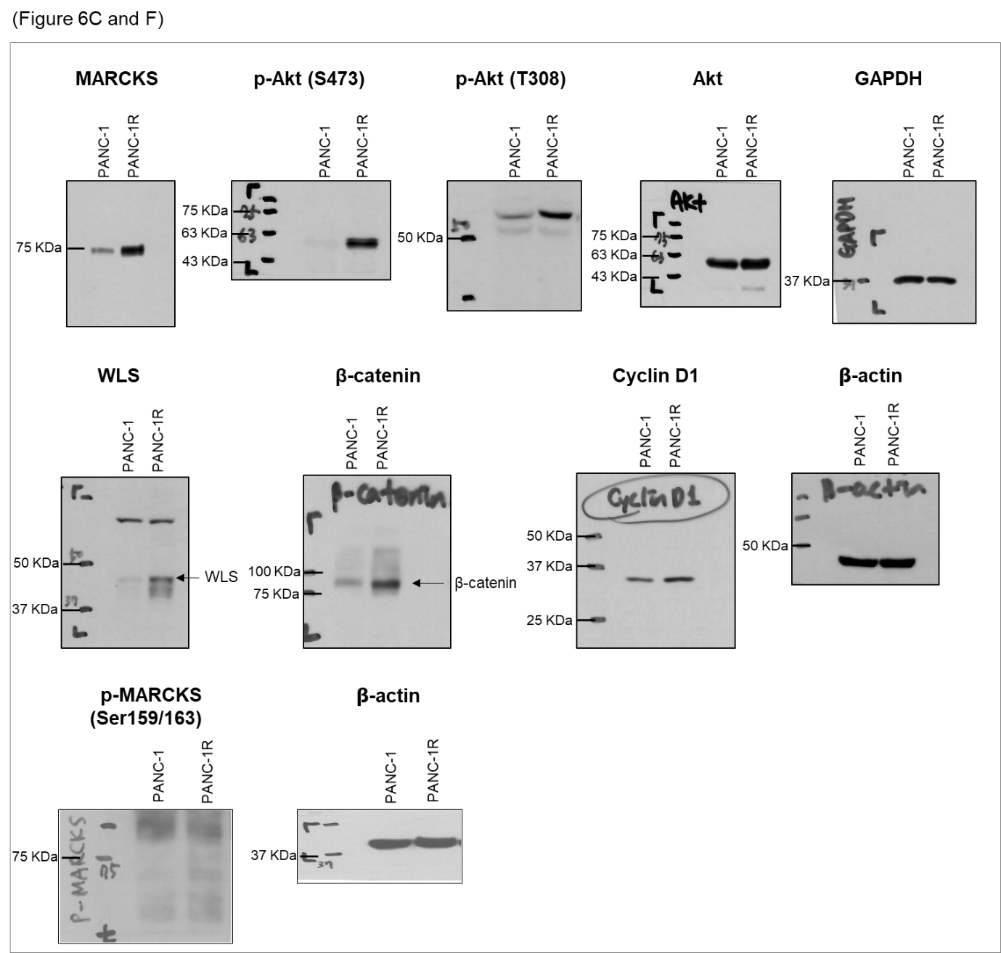


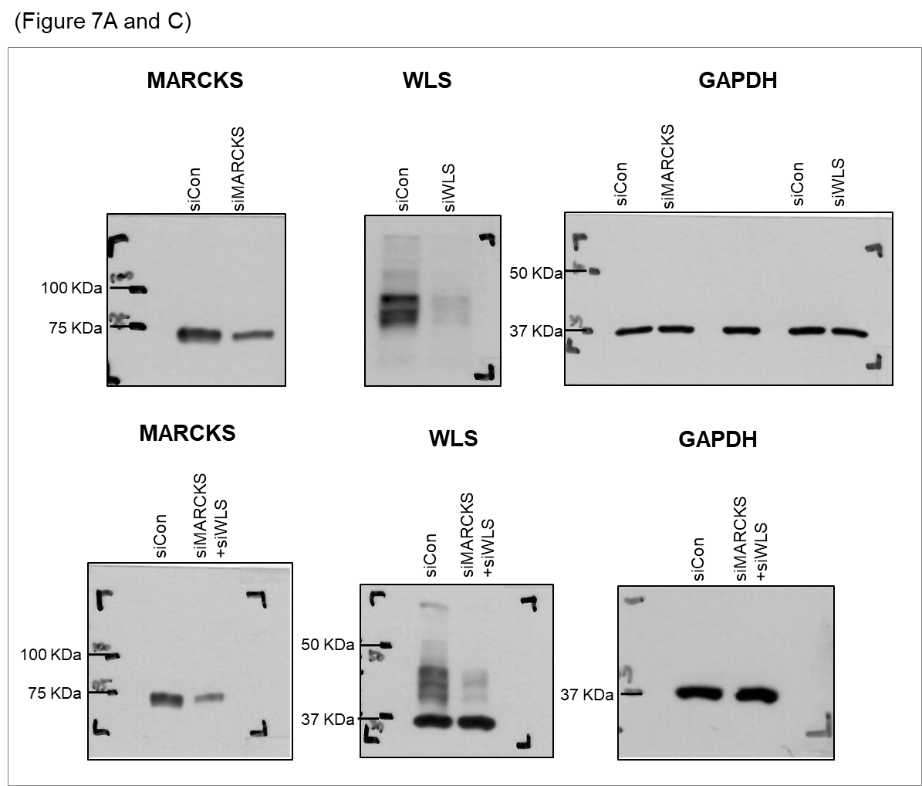


**Figure S2.** Full-length western blot images for Figure 5A, 6C, 6F, 7A and 7C in the main text.


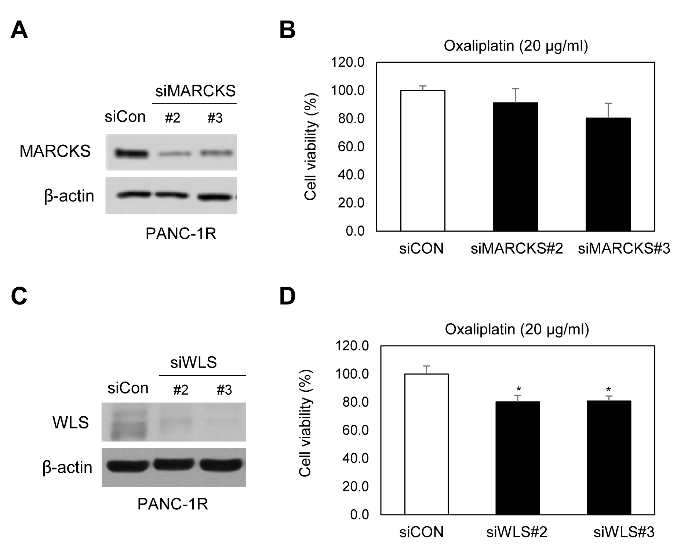


**Figure S3.** The suppression of MARCKS or WLS by siRNA affected the sensitive to oxaliplatin in PANC-1R cells. (A) The level of MARCKS with the treatment of siCON, siRNA #2 and #3. (B) The cell viability to oxaliplatin was analyzed by Ex-cytox in PANC-1R with knockdown of MARCKS. (C) The level of WLS with the treatment of siCON, siRNA #2 and #3. (D) The cell viability to oxaliplatin was analyzed by Ex-cytox in PANC-1R with knockdown of WLS. * *p* < 0.05.
